# Unpacking associations among children’s spatial skills, mathematics, and arithmetic strategies: decomposition matters

**DOI:** 10.1007/s00426-024-01952-x

**Published:** 2024-04-13

**Authors:** Wenke Möhring, Léonie Moll, Magdalena Szubielska

**Affiliations:** 1 Faculty of Psychology, Department of Psychology, University of Basel, Missionsstrasse 62, 4055 Basel, Switzerland; 2 Department of Educational and Health Psychology, University of Education Schwäbisch Gmünd, Schwäbisch Gmünd, Germany; 3 Institute of Psychology, Faculty of Social Sciences, The John Paul II Catholic University of Lublin, Lublin, Poland

## Abstract

Several studies revealed links between mental rotation and mathematical tasks, but the intervening processes in this connection remain rather unexplored. Here, we aimed to investigate whether children’s mental rotation skills relate to their accuracy in solving arithmetic problems via their usage of decomposition strategies, thus probing one potential intervening process. To this end, we examined a sample of 6- to 8-year-olds (*N* = 183) with a chronometric mental rotation task, and asked children to solve several arithmetic problems while assessing their solution strategies. After each arithmetic problem, children were asked about their strategy to solve the respective arithmetic problem and these were classified as either counting, decomposition, or retrieval strategies. Analyses were controlled for age, sex, fluid and verbal reasoning. Results indicated that children’s response times and accuracy in the mental rotation task were best explained by linear functions of rotation angle, suggesting the usage of dynamic mental transformation strategies. A multiple mediation model revealed that children with higher mental rotation skills were more inclined to use higher-level mental strategies such as decomposition which in turn increased their accuracy of solving arithmetic problems. None of the other arithmetic strategies revealed significant indirect effects. These findings suggest that children with higher mental rotation skills may profit from visualizing and flexibly transforming numerical magnitudes, increasing the frequency of decomposition strategies. Overall, decomposition may play a unique role in the connection between children’s mental rotation and arithmetic skills, which is an essential information for planning future training and experimental studies.

## Introduction

Spatial ability is typically defined as being able to generate, retain, retrieve, and transform mental visual images (Lohman, 1996). This ability plays an important role for educational success (Newcombe et al., 2018) and was predictive for pursuing a career in Science, Technology, Engineering, and Mathematics (STEM) disciplines. For example, in a large-scale study based on a sample of more than 400′000 adolescents, it was found that spatial ability in grade 9–12 predicted later career choices in STEM-related fields, even after controlling for other cognitive abilities (Wai et al., 2009). This result underlines the unique role of spatial thinking for *entering* STEM fields but other studies indicated also predictions of professional *success* in STEM disciplines (Kell et al., 2013). With respect to children, several studies have shown that spatial thinking relates particularly to children’s mathematical achievement (e.g., Judd & Klingberg, 2021; Lauer & Lourenco, 2016; Möhring et al., 2021; Verdine et al., 2017; for meta-analyses, see Atit et al., 2022; Xie et al., 2020). This result may not come as a surprise given that several mathematical themes such as geometry are inherently spatial. However, evidence for children’s spatial-numerical relations was also found for topics such as calculating with symbolic Arabic numbers (e.g., Frick, 2019; Gunderson & Hildebrand, 2021; Gunderson et al., 2012; Moè, 2018).

Several studies have operationalized spatial thinking by measuring children’s performance in a mental rotation task (e.g., Carr et al., 2017; Casey et al., 1995; Kyttälä & Lehto, 2008; Mix et al., 2016; for a review, see Mix & Cheng, 2012). Mental rotation is a prototypical spatial skill and refers to participants’ ability to mentally represent an object and to transform this mental image (for a review, see Frick et al., 2014). According to a recent classification model (Newcombe & Shipley, 2015; Uttal et al., 2013), mental rotation involves processing the intrinsic information within an object (i.e., the relations between parts of a single object), as well as being able to dynamically transform the entire object. Following this classification model, spatial skills can also involve processing static or extrinsic information (i.e., relations between an object and other surrounding objects). However, the majority of studies investigating relations between spatial and mathematical skills has focused on intrinsic, dynamic spatial skills like mental rotation (Young et al., 2018). Moreover, Mix et al. (2016) showed that mental rotation showed specific cross-loadings on a spatial and mathematical factor in a factor analysis, so that the authors concluded that certain spatial skills may have a particularly strong relation with mathematics.

In a typical mental rotation task (Shepard & Metzler, 1971), adults are presented with two objects in different orientations and are asked to decide whether these objects are identical or not. In some trials, objects are the same and can be superimposed by mentally rotating one object into the same position as the other object, whereas in other trials, one object is a mirror image and can never be superimposed. Using different rotation angles, it was found that participants’ response times (RTs) and errors in these same-different decisions increased linearly with larger angles. This linear function has been interpreted in favor of analog mental representations (i.e., mental images) in the human mind (Kosslyn, 1975; Kosslyn et al., 1990), which can be dynamically transformed. This respective task has been successfully adapted to even younger age groups and studies indicated that children as young as 5–6 years can successfully rotate objects in their minds (e.g., Estes, 1998; Marmor, 1975; Perrucci et al., 2008; for a review, Frick et al., 2014), with easier tasks indicating early beginnings in infancy (Hespos & Rochat, 1997; Möhring & Frick, 2013; Moore & Johnson, 2008; Quinn & Liben, 2008; Schwarzer et al., 2013).

Several studies indicated a close connection between children’s mental rotation and mathematical achievement (e.g., Carr et al., 2017; Casey et al., 1995; Kyttälä & Lehto, 2008; Mix et al., 2016; for a review, see Mix & Cheng, 2012). This result has led to speculations that spatial thinking and in particular mental rotation might be harnessed to improve mathematical thinking. Studies have begun to conduct spatial trainings and investigated far transfer to mathematical learning (e.g., Gilligan et al., 2020; Lowrie et al., 2017). A recent meta-analysis has summarized these intervention studies and yielded beneficial effects of spatial training on children’s mathematical achievement (Hawes et al., 2022). This outcome supports a causal relation between spatial and mathematical skills. Further support for this causal relationship comes from cross-lagged panel models indicating that *early* spatial skills predict *later* mathematical abilities whereas a similar, reversed relation seems unsupported (Fung et al., 2020; Kahl et al., 2022, but see Geer et al., 2019).

In light of these studies, it seems crucial and timely to increase our understanding about the mechanisms underlying this link between space and math (Lowrie et al., 2020; Mix, 2019). As spatial trainings were often broadly conceptualized, it is hard to pinpoint the critical factors that help improving mathematical skills. Of course, the ideal way to investigate causality requires an experimental methodology (Bailey, 2017). However, a first step may refer to examining potential intervening processes in this space-math relation. An increased knowledge about these intervening processes is crucial for planning subsequent experimental and longitudinal approaches. Moreover, findings will yield important implications for theory and practice by for instance informing educational policies or enriching spatial trainings.

One intervening process that has been outlined in previous research referred to children’s representations of numbers on a mental number line (Gunderson et al., 2012). In her original study, Gunderson and colleagues showed relations between 5-year-olds’ mental transformations skills and their symbolic calculation skills at age 8. Importantly, this relation was fully mediated by children’s number line estimations at age 6 (Gunderson et al., 2012). The authors concluded that spatial skills may help children to form a meaningful numerical representation, in which numbers are linearly ordered from small to large. Given that this respective study has used a rather small sample (*N* = 42), several follow-up studies tried to replicate this finding but failed to show this mediation (e.g., Frick, 2019; Gunderson & Hildebrand, 2021; LeFevre et al., 2013). Therefore, at least until now, this intervening process between space and math via number line representations remains rather unsupported.

## Arithmetic strategies: decomposition matters?

Another potential candidate that has been proposed in previous studies might be children’s strategy use (for a review, see Casey & Fell, 2018; Casey et al., 2017; Foley et al., 2017; Laski et al., 2013). When children solve calculation problems, they may choose from a variety of strategies, ranging from counting strategies to higher-level mental strategies such as decomposition and fact retrieval from memory. Decisions about strategy use depend on children’s knowledge and current task demands, and reflect children’s beliefs about which strategy may yield the greatest accuracy for this respective problem (Shrager & Siegler, 1998).

Several strategies have been differentiated in previous studies (Casey et al., 2017; Laski et al., 2013; Ramirez et al., 2016). For example, children may count each addend of an arithmetic problem (e.g., 5 + 2) and then count the total (e.g., they count to 5 and then count to 2, and finally count all the numbers from 1 to 7). Such a *count-all* strategy is very time intensive. Another quicker counting strategy refers to the *count-on* strategy, in which children count one addend and then add the numerical value of the other addend (e.g., they count to 5, and then count 6, 7 to solve the same problem as above). Counting strategies are helpful when computing problems with small numbers (< 10), which can be easily solved using our fingers. However, as soon as numbers exceed 10, counting strategies become less efficient and more error-prone. Consequently, one of the major mathematical accomplishments during early primary school is that children learn to overcome these relatively simple counting strategies towards implementing more advanced mental strategies (Carr & Alexeev, 2011). One example of a higher-level mental strategy refers to fact retrieval. When using this strategy, children would mentally recall the result from memory because they learnt the result by heart. Another higher-level mental strategy refers to decomposition. When using this respective strategy, a child would simplify a mathematical problem by decomposing a number into smaller parts and taking multiple steps to solve the problem. Such a decomposition strategy can build upon the base-10 properties of the number system (e.g., when changing 5 + 8 to 5 + 5 + 3). But decomposition can also be based upon previously learnt number facts when using identical addends (when changing 6 + 8 to 6 + 6 + 2) or when transforming the problem into a better-known problem (when changing 12 + 7 to 12 + 3 + 4; Laski et al., 2014). These decomposition strategies can be used with several types of arithmetic problems but the probability of choosing this strategy increases when problems involve larger number (Laski et al., 2014). Moreover, children use more likely base-10 decomposition strategies when problems require a decade change (Laski et al., 2014).

Previous studies have revealed close links between these decomposition strategies and spatial skills including mental rotation tasks (e.g., Laski et al., 2013). Moreover, decomposition in 1st grade was closely related to later mathematical understanding (Carr & Alexeev, 2011; Geary, 2011). Importantly, research showed that children’s spatial skills predicted their later mathematical abilities via their usage of decomposition strategies, thus indicating an intervening pathway via decomposition (Casey et al., 2017; visual-spatial working memory: Foley et al., 2017). In the study from Casey et al. (2017), the authors used a decomposition task (i.e., the block design task from the WISC-IV) as well as two mental rotation tasks. All of these tasks tap intrinsic-dynamic spatial abilities in accordance to the classification model from Newcombe and Shipley (2015) and Uttal et al. (2013). Building upon these findings, it seems that spatial skills may help representing numerical magnitudes and allow children to dynamically segment these large magnitudes into smaller parts. Following this line of argumentation, spatial skills may help visualizing numerical magnitudes (i.e., 8), help segmenting and transforming these magnitudes (e.g., splitting the same magnitude into 3 and 5), which results in higher frequencies of decomposition strategies. Ultimately, these visualization and transformation processes help increasing mathematical performance.

These studies are important starting points and support the notion of decomposition strategies being an intervening pathway connecting spatial and mathematical skills. However, most of these studies were exclusively conducted with girls (Casey et al., 2017; Laski et al., 2013), or with relatively small samples (*N* = 78; Foley et al. 2017). Consequently, it remains unclear whether decomposition is a critical intervening process interlinking *boys’* spatial and mathematical reasoning and thus, might be a universal intervening process. Second, important control variables such as fluid reasoning were missing in these previous studies (for a discussion, cf. Casey and Ganley 2021), which seems important given close associations between fluid reasoning, spatial and mathematical skills (e.g., Atit et al., 2022; Peng et al., 2019).

## The present study

Building upon this research, in the present study, we aimed to investigate whether children’s mental rotation skills relate to their accuracy in solving arithmetic problems via their usage of decomposition strategies. To this end, we examined a sample of 6- to 8-year-olds (*N* = 183) with a classic chronometric mental rotation task. We used particularly a mental rotation task for two reasons: On the one hand, it is a prototypical spatial task which was used rather extensively in previous research on the topic (e.g., Casey et al., 1995; Frick, 2019; Mix et al., 2016), enabling comparability among different studies. On the other hand, this task allows investigating whether children transform their mental visual images dynamically. In line with mental rotation research (Kosslyn, 1975; Kosslyn et al., 1990; Shepard & Metzler, 1971), we expected children’s RTs and errors to be linear functions of rotation angle which would support dynamic transformation strategies.

In addition, we asked children to solve several arithmetic problems and assessed their solution strategies. To this end, after each arithmetic problem, children were asked about their strategies. Children’s explanations as well as their overt behavior was used to classify strategies in line with previous research (Laski et al., 2013; Ramirez et al., 2016). To answer the research question whether mental rotation skills correlated with children’s arithmetic problem solving via decomposition, we computed a multiple mediation model, in which all arithmetic strategies were entered simultaneously. This approach enabled examining the uniqueness of each mediator in the relation between mental rotation and arithmetic skills. In accordance of previous research (Casey et al., 2017; Foley et al., 2017), we expected to see relations between children’s mental rotation and arithmetic skills via their usage of decomposition. We expected to see this indirect effect at least in girls, but additionally explored the same effect in boys. Analyses were controlled for age, sex, verbal and fluid reasoning. Previous studies suggested differential relations between spatial and mathematical skills for girls and boys (e.g., Klein et al., 2010). Therefore, in an additional set of analyses, we checked whether the relations among mental rotation, arithmetic skills and strategies differed between males and females by adding interaction terms to the multiple mediation model.

## Methods

### Participants

A total of 183 6- to 8-year-olds participated in the present study (57.4% male, 85.2% right-handed,^1^ for descriptive variables, see Table 1). Twenty additional children were tested but had to be excluded due to technical error with the software (*n* = 3), because they performed lower than chance level in the mental rotation task (*n* = 13), or did not take part in the arithmetic task (*n* = 4). Children attended Swiss and German kindergartens and schools. The majority of children was White. Participants were excluded when they were diagnosed with developmental disorders such as autism-spectrum disorder and when the children’s level of German language skills was not fluent enough to ensure that they understood the task instructions. The ethics committee of the respective University approved the current study (protocol number: 004-21-1). Children provided verbal assent prior to study participation and parents gave written informed consent. Children and their families received a voucher for their participation.

**Table 1:** Descriptive statistics of all study variables (*N* = 183)

	*M* (*SD*)	Min	Max
Mean age	7.55 (0.71)	6.02	8.99
WISC-V vocabulary (raw)^1^	24.68 (6.03)	5	35
WISC-V vocabulary (standardized)^2^	11.96 (3.10)	1	19
WISC-V matrices (raw)^1^	15.40 (3.75)	5	26
WISC-V matrices (standardized)^2^	10.97 (2.47)	3	18
Mental rotation			
# correct	50.91 (6.72)	28	56
Response times (in ms)	2554 (518)	855	3902
Arithmetic task			
# correct	13.70 (5.69)	0	20
Arithmetic strategies			
Count-all	0.45 (1.76)	0	15
Count-on	3.60 (5.10)	0	20
Decomposition	7.49 (6.32)	0	20
Retrieval	3.75 (4.30)	0	20
Guess	0.38 (0.99)	0	6
Using concrete material or fingers in arithmetic task			
Concrete material (pearls)	0.38 (1.65)	0	17
Fingers	2.97 (4.87)	0	19

^1^refer to raw scores in each subtest

^2^refer to age-standardized scores with *M* = 10, *SD* = 3

Standard deviations (*SD*) are presented in parentheses

### Power analysis

When computing an a priori power analysis using G-Power 3.1 (Faul et al., 2007), we assumed a low-to-moderate effect size for an indirect effect of decomposition in a mediation analysis based on previous research (cf. Casey et al., 2017). Using this effect size, a two-tailed significance level of *p* < . 05, a statistical power of 0.85, and a total of 9 predictors in a regression model (mental rotation, arithmetic strategies, and control variables), a sample of 163 children would be required for sufficient power. Therefore, it seems that our sample was adequately powered to address the present research question.

### Measures

Children were tested individually within a single experimental session by trained research assistants. They were examined in a quiet, separate room at their educational institution or in a laboratory at the respective University. Children took part in several tasks during this testing session, with some of these tasks being outside the scope of the present study.^2^ A description of the tasks that are not central to the present research question, can be found in the respective publications (Möhring & Szubielska, 2023).

*Fluid and verbal reasoning.* To assess children’s typical cognitive development, children were presented with two subtests from the Wechsler Intelligence Scale for Children-5th Edition, German version (WISC-V, Petermann, 2017; Wechsler, 2014; age range: 6–16 years). The WISC-V is a well-acknowledged instrument assessing several components of intellectual ability and complying high reliability and validity standards (for a discussion, see Canivez & Watkins, 2016). Children were examined with the subtests “vocabulary” and “matrix reasoning” given that performances on these subtests are typically seen as proxies for children’s verbal and fluid intelligence respectively (Groth-Marnat, 2009).

*Mental rotation.* Children were presented with a chronometric mental rotation task on a computer screen using Cedrus Superlab 4.5 software (cf. Estes, 1998; Marmor, 1975). In accordance with previous studies using same-aged samples, children saw pictures of two panda bears raising an arm (cf. Marmor, 1975; Perrucci et al., 2008). One bear was rotated in the picture plane with different rotation angles being used (ranging from 0 to 180°, in 30° steps) whereas the other bear stood upright. The panda bears on the display were either identical or mirror images. Children were asked to decide whether both panda bears raised the same or different arms by pressing one of two computer keys. A manipulated keyboard that contained only two keys, one on the left and one on the right side, was located in front of the monitor. One key was assigned as the “same” key; the other key was assigned as the “different” key. Little pictures symbolizing “same” (i.e., presenting two identical blue triangles) and “different” (i.e., presenting a blue triangle and a red circle) were glued above each key and acted as reminders of these labels throughout the experimental session.

Furthermore, it was systematically manipulated whether the left (L) or right (R) panda bear stood upright, with order being counterbalanced between children. Approximately half of the participants was presented with the order LRRL (*n* = 95); the other half was presented with RLLR (*n* = 88). Test trials were preceded by a short pre-training to familiarize children with the task. Using six practice trials, children were introduced to the task with different angles (i.e., 45° and 135°) as compared to later test trials. Children received feedback after each response in these practice trials (e.g., smileys with a happy face). Test trials proceeded immediately afterwards but provided no feedback. Using a full-factorial design, these within-participant variables of angle (from 0 to 180°, in 30° steps; 7), side of rotation (left vs. right; 2), answer type (same vs. different, 2) and repetition (2) amounted to a total of 56 trials. Test trials were presented in randomized order. Children were invited to take a short break after the first half of the trials (28 trials). The number of correct answers and RTs from correctly solved trials served as dependent variables (for an analogous procedure, see Perrucci et al., 2008).

*Mathematical task assessing arithmetic strategies.* Children were presented with a total of 20 arithmetic problems involving one-digit and two-digit numbers, with half of them involving a decade change (e.g., 18 + 5, see Appendix). These arithmetic problems were presented in a fixed order and increased in difficulty. At the beginning of the task, children were presented with two simple addition problems involving one-digit numbers (e.g., 5 + 3). Afterwards, children were presented with eight addition problems involving a one-digit and a two-digit number (e.g., 9 + 26). Children were then presented with five addition problems involving three addends (e.g., 4 + 3 + 1) and five missing-term problems (e.g., 4 + _ = 5 + 3), which alternated for the second half of the task. Overall, the majority of problems involved addition skills, except of the missing term problems which required also subtraction.

Prior to solving these problems, children were instructed that they would see mathematical problems on the computer screen and were asked to solve them. They were told that they can solve them however they like and can even use some wooden pearls, presented next to the computer, if this would be helpful. The task started with four practice trials to acquaint children with the task. These practice trials involved only one-digit numbers and represented each type of question (e.g., 2 + 3, 1 + 1 + 7, 1 + _ = 2 + 3). With respect to the missing term problem, the experimenter explained the meaning of these symbols (e.g., _ as an indicator that a number is missing as well as the equal sign) to make sure that all children understood this problem type. Afterwards, the test trials were started and children were presented sequentially with each arithmetic problem. The number of correct responses served as dependent variable.

After their response, children were asked how they solved each problem. These verbal explanations but also their overt behavior (such as finger counting) were used to classify the arithmetic strategies. Research assistants were trained with respect to classifying arithmetic strategies, and carefully noted whether children used their fingers or the wooden pearls, but also the exact computation that children explained verbally. For example, a strategy was classified as decomposition when the child reported segmenting a numerical magnitude into smaller parts and taking multiple steps. A strategy was classified as count-all, when the child obviously counted one number (often using one hand) and then counted the other one (often using the other hand), before finally counting the total number. If children provided an inconsistent explanation with their overt behavior (e.g., used finger counting and explained that they just knew the answer), their overt behavior was used to code the strategy (e.g., Laski et al., 2013; Ramirez et al., 2016). If children reported they had just guessed or did not know the answer, these responses were classified as guess. Guesses were not considered in any analyses. Similar to previous research (Laski et al., 2013; Ramirez et al., 2016), the number of using each arithmetic strategy (count-all, count-on, decomposition, retrieval) in the arithmetic problems served as variables in the statistical models.

### Statistical analyses

Analyses were conducted using IBM SPSS 27. To investigate whether children used mental transformation strategies in the mental rotation task, we first assessed effects of rotation angle using repeated measures analyses of variance (ANCOVAs) with rotation angle as within-participant variable, sex and condition (LRRL, RLLR) as between-participants variables, and age as a continuous covariate. Children’s correct answers and RTs in each rotation angle served as dependent variables. Following previous mental rotation research (for an overview, see Frick et al., 2014; Kosslyn, 1975; Kosslyn et al., 1990), we were particularly interested in seeing whether these dependent variables varied linearly as a function of rotation angle. Greenhouse–Geisser corrections were used to account for violations of the sphericity assumption whenever necessary. Significant effects in ANCOVAs were followed up by post hoc comparisons using Sidak adjustments.

Mediation analyses were computed with the PROCESS plug-in version 4.1 from Hayes (2018, model 4), using the bootstrapping method with 5,000 samples and 95% confidence intervals (CI). Children’s overall correct answers in the mental rotation task served as predictor variable, the number of correct answers in the mathematical task was entered as criterion, and arithmetic strategies served as mediating variables. Sex, age, fluid and verbal reasoning were entered as control variables. Arithmetic strategies were entered simultaneously in the multiple mediation model and all variables (except sex) were standardized before computing the multiple mediation model to assure that scales were comparable.

## Results

### Descriptive statistics and bivariate correlations among the study variables

An outlier analysis for RTs in the mental rotation task revealed that 1.81% of all responses exceeded the criteria of mean ± 3 *SD*s and below 120 ms (Ishihara et al., 2006; Möhring et al., 2019). These values were excluded. Children’s accuracies and RTs were collapsed for each rotation angle.

Descriptive statistics (see Table 1) revealed that children solved several items in the mental rotation and arithmetic task correctly. Yet, standard deviations yielded considerable interindividual variation in children’s performance. With respect to arithmetic strategies, it was found that children used rarely count-all strategies, and relied predominantly on decomposition strategies, followed by count-on and retrieval strategies. Additionally, children used seldomly concrete material (i.e., wooden pearls) but relied more often on using their fingers to support their mental calculations.

The bivariate correlations (see Table 2) yielded close associations between mental rotation and arithmetic skills, even after accounting for age, sex, fluid and verbal reasoning. Mental rotation correlated also positively with decomposition strategies. Children’s usage of decomposition and retrieval strategies correlated positively with the number of correctly solved arithmetic problems. Conversely, their usage of count-all strategies correlated negatively with accuracy in the arithmetic task. Count-on strategies correlated negatively with using higher-level mental strategies such as decomposition and retrieval. A look at potential control variables revealed that fluid and verbal reasoning were associated with a number of the variables of interest such as accuracy in the arithmetic task or mental rotation, highlighting the importance to include those variables in the statistical models. Similarly, age was related to a number of variables such as strategy use and arithmetic skills, suggesting that older children were more inclined to use higher-level mental strategies and solved more arithmetic problems correctly. Finally, there were several correlations with sex, suggesting that females used more often count-on strategies, and less likely decomposition and retrieval strategies.

**Table 2:** Bivariate correlations among the variables of interest (*N* = 183)

	(1)	(2)	(3)	(4)	(5)	(6)	(7)	(8)	(9)	(10)	(11)
1. Mental rotation		.26***	− .08	− .07	.21**	.07	− .28***	.09	.16*	.09	− .05
2. Arithmetic skills	.22**		− .34***	− .001	.59***	.47***	− .25***	.35***	.51***	.68***	− .13
3. Arithmetic strategy: count-all	− .04	− .19*		− .07	− .26***	− .15*	.02	− .26***	− .23**	− .27***	− .07
4. Arithmetic strategy: count-on	− .03	.20**	− .10		− .40***	− .31***	.07	.05	− .17*	− .12	.31***
5. Arithmetic strategy: decomposition	.16*	.37***	− .13	− .38***		.03	− .18*	.33***	.38***	.48***	− .18*
6. Arithmetic strategy: retrieval	.02	.24**	− .05	− .24**	− .28***		− .17*	.10	.30***	.43***	− .20**
7. Arithmetic strategy: guess	− .27***	− .14	− .10	.10	− .10	− .12		− .11	− .28***	− .20**	− .22**
8. Verbal reasoning (WISC-V, raw)									.27***	.37***	.02
9. Fluid reasoning (WISC-V, raw)										.45***	.02
10. Age											− .13
11. Sex											

Above diagonal: zero-order correlations; below diagonal: partial correlations, controlled for age, sex, fluid and verbal reasoning

Sex: female = 1, male = − 1. ∗  ∗  ∗ *p* < 0.001, ∗  ∗ *p* < 0.01, ∗ *p* < 0.05

Significant effects are highlighted in bold

### Mental rotation: effects of rotation angle

**Fig. 1:**
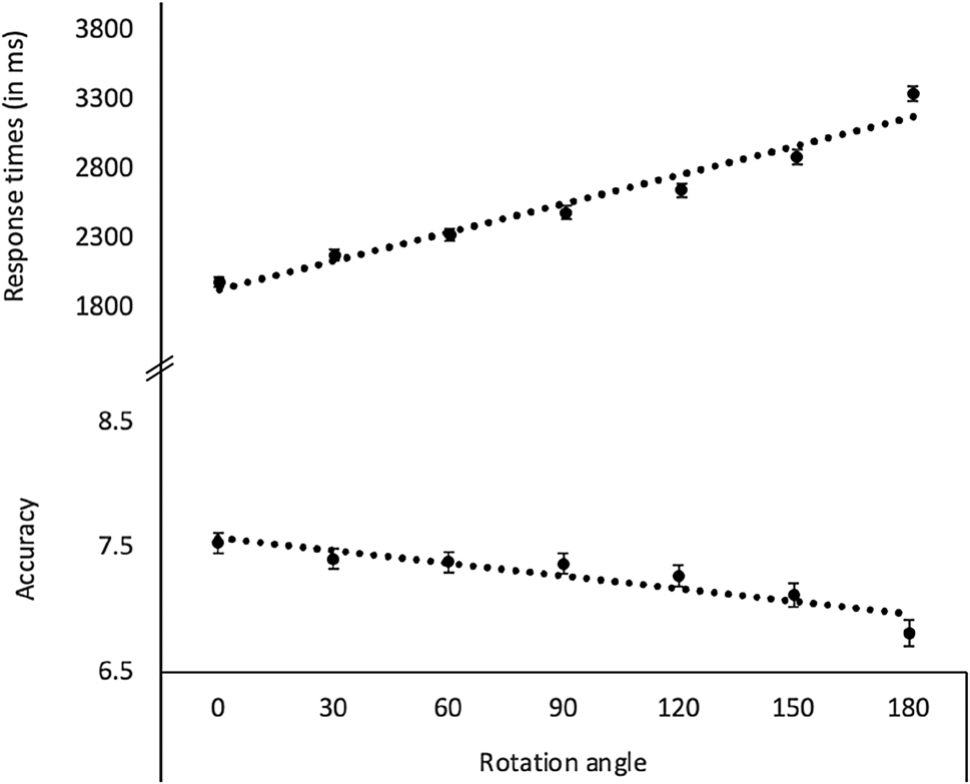
Children’s accuracy and response times as a function of rotation angle in the mental rotation task. Error bars indicate ± 1 standard errors

The ANCOVAs investigating effects of rotation angle revealed a significant effect of rotation angle on children’s accuracy and a marginal significant effect on their RTs (for descriptive and inferential statistics, refer to Table 3). As can be seen in Fig. 1, children showed a decreasing accuracy and higher RTs when rotation angles became larger. Both effects were best explained by a linear function (accuracy: *F*(1, 178) = 5.92, *p* < 0.05, η_P_^2^ = 0.03; RTs: *F*(1, 177) = 7.74, *p* < 0.01, η_P_^2^ = 0.04). The ANCOVAs revealed no effects of age, sex, nor condition on children’s accuracy. However, for children’s RTs, there was a significant age effect because older children responded quicker as opposed to younger children (Bs ranged from − 215 to − 264 for RTs in different angles). Finally, the same ANCOVA yielded a significant interaction between condition and sex. However, follow-up pairwise comparisons revealed that females and males did not differ with respect to RTs in both conditions (both *p*s > 0.095).

**Table 3:** Descriptive and inferential statistics of the ANCOVAs investigating effects of rotation angle on children’s accuracy and RTs

	Accuracy				RTs			
	*dfs*	*F*	*P*	*η*_*P*_^*2*^	*Dfs*	*F*	*p*	*η*_*P*_^*2*^
Rotation angle	**4.380, 779.602**	2.451*	**.040**	**.014**	4.990, 883.279	2.028	.073	.011
0	7.536 (1.093)				1987 (521)			
30	7.410 (1.054)				2182 (532)			
60	7.383 (1.100)				2329 (583)			
90	7.372 (1.116)				2491 (615)			
120	7.273 (1.135)				2650 (644)			
150	7.120 (1.300)				2892 (736)			
180	6.814 (1.417)				3346 (751)			
Age	1, 178	1.147	.286	.006	**1, 177**	20.734***	** < .001**	**.105**
Condition	1, 178	1.020	.314	.006	1, 177	0.005	.945	< .001
LRRL	51.411 (5.913)				2533 (488)			
RLLR	50.364 (7.487)				2576 (550)			
Sex	1, 178	0.373	.542	.002	1, 177	0.075	.785	< .001
Female	50.500 (7.406)				2601 (462)			
Male	51.210 (6.176)				2519 (555)			
Angle × age	4.380, 779.602	1.628	.159	.009	4.990, 883.279	0.166	.975	.001
Angle × condition	4.380, 779.60	0.967	.430	.005	4.990, 883.279	0.453	.811	.003
Angle × sex	4.380, 779.60	1.178	.319	.007	4.990, 883.279	0.466	.802	.003
Condition × sex	1, 178	0	.993	0	**1, 177**	4.05**	**.046**	**.022**
Angle × condition × sex	4.380, 779.60	0.341	.866	.002	4.990, 883.279	0.660	.654	.004

*** *p* < .001, ** *p* < .01, * *p* < .05

Standard deviations (*SD*) are presented in parentheses

Significant effects are highlighted in bold

The analysis with RTs was based on only *n* = 182 children because one child had too many missing values for the rotation angle 180

### Mediation analysis with strategy use

The total effect model revealed that mental rotation as well as the control variables were able to explain 54.4% of the total variance in children’s arithmetic skills (see Table 4), and thus, more than half of the individual variation in children’s accuracy to solve arithmetic problems. Furthermore, the same model revealed that mental rotation and accuracy in solving the arithmetic problems were highly related with each other, *β* = 0.161, *p* < 0.01. Therefore, it seems that children with higher mental rotation skills solved more arithmetic problems, even after accounting for age, sex, fluid and verbal reasoning.

**Table 4:** Mediation analyses (*N* = 183) with strategy use as mediating variables between mental rotation and arithmetic skills

Model	Estimate	*SE*/*SE*^*t*^	*p*	R^2^	95% CI
Model without mediator					
Intercept	− .009	.051	.854		(− .110, .092)
Mental rotation → Arithmetic skills (c)	.161**	**.052**	** < .01**	.544***	**(.059, .263)**
Model with all mediators					
Intercept	− .008	.036	.830		(− .079, .063)
Mental rotation → Arithmetic skills (c’)	.087*	**.037**	** < .05**	.781***	**(.014, .159)**
Model with count-all as mediator					
Intercept	− .014	.071	.849		(− .154, .127)
Mental rotation → Count-all (a)	− .035	.072	.625	.121***	(− .176, .106)
Count-all → Arithmetic skills (b)	− .006	.039	.880		(− .083, .071)
Indirect effect (a*b)	0	.004^*t*^			(− .004, .012)
Model with count-on as mediator					
Intercept	.046	.070	.512		(− .093, .185)
Mental rotation → Count-on (a)	− .030	.071	.671	.139***	(− .170, .110)
Count-on → Arithmetic skills (b)	.450***	**.046**	** < .001**		**(.360, .540)**
Indirect effect (a*b)	− .014	.038^*t*^			(− .099, .052)
Model with decomposition as mediator					
Intercept	− .020	.063	.747		(− .144, .103)
Mental rotation → Decomposition (a)	.135*	**.063**	** < .05**	.317**	**(.010, .259)**
Decomposition → Arithmetic skills (b)	.604***	**.052**	** < .001**		**(.501, .707)**
Indirect effect (a*b)	.081*	**.039**^***t***^			**(.016, .171)**
Model with retrieval as mediator					
Intercept	-.023	.067	.736		(-.154, .109)
Mental rotation → Retrieval (a)	.015	.067	.828	.224***	(− .118, .147)
Retrieval → Arithmetic skills (b)	**.457**	**.046**	** < .001**		**(.366, .549)**
Indirect effect (a*b)	.007	.031^*t*^			(-.058, .068)

***p < .001, **p < .01, *p < .05. SE = standard errors. *SE*^*t*^ refers to bootstrapped standard errors, based on 5000 samples. Values that are *SE*^*t*^ are denoted with the same symbol

*CI *Confidence interval

Analyses were controlled for age, sex, fluid and verbal reasoning. Significant effects are highlighted in bold

The regression models assessing relations between mental rotation and arithmetic strategies showed that mental rotation was significantly correlated with decomposition strategies, but not with any other arithmetic strategy. Thus, it seems that after accounting for effects of age, sex, fluid and verbal reasoning, children with higher mental rotation skills were more inclined to decompose numbers and simplify arithmetic problems. Furthermore, in the model in which mental rotation and arithmetic strategies were entered simultaneously as predictor variables (in addition to the control variables), it was found that the explained variance in children’s accuracy of solving arithmetic problems increased to 78.1%. Therefore, adding arithmetic strategies increased the explained variance considerably as opposed to the total model. This regression model also showed that all arithmetic strategies except of the count-all strategy were positively related to children’s accuracy in solving arithmetic problems. Moreover, mental rotation and arithmetic skills were still significantly related to each other, even though the standardized regression coefficient became smaller (*β* = 0.087, *p* < 0.05), when accounting for arithmetic strategies in the same model.

**Fig. 2:**
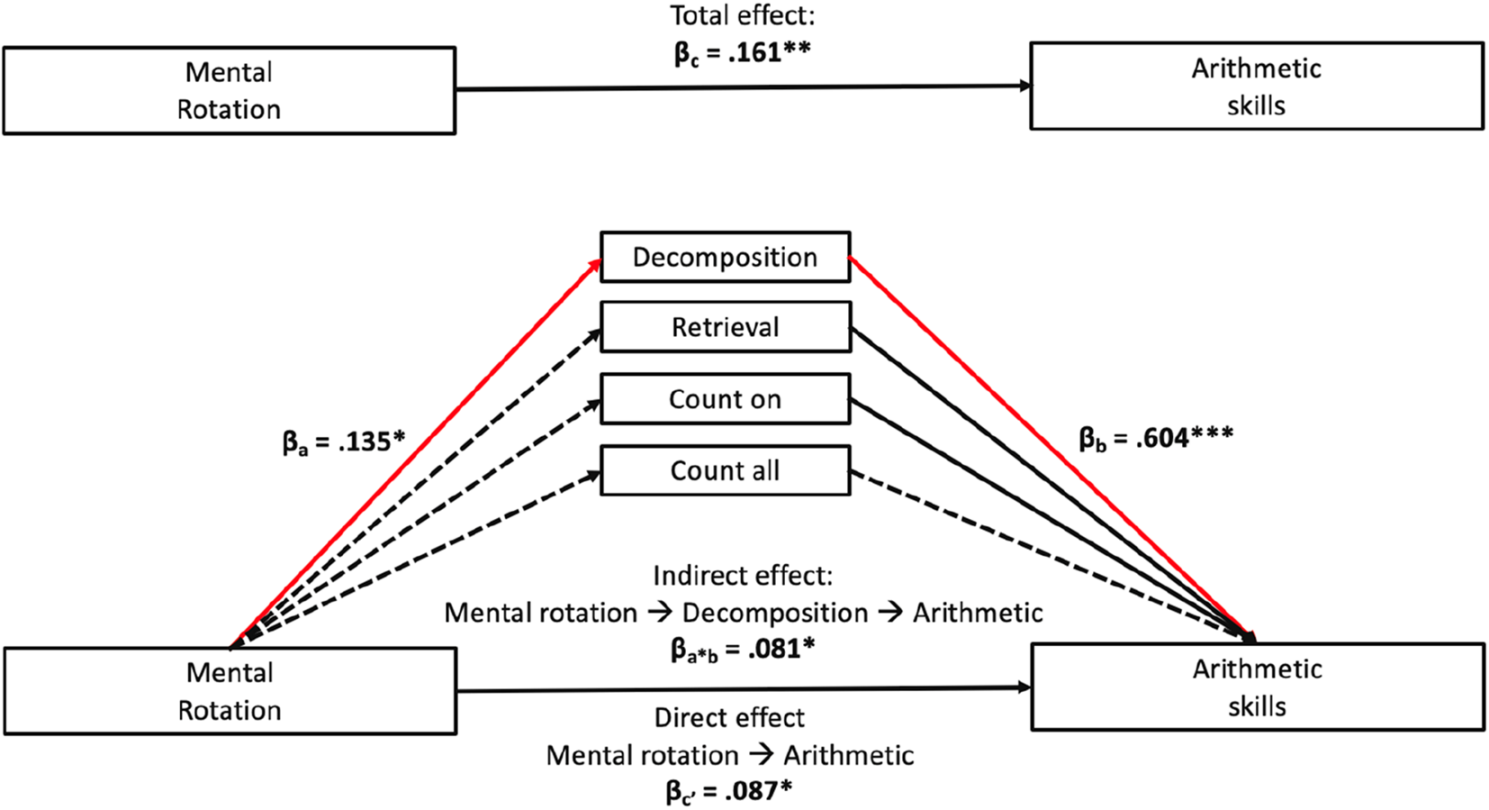
Mediation analyses with strategy use as mediating variables between mental rotation and arithmetic skills (*N* = 183). Dashed lines indicate non-significant relations, solid lines indicate significant relations. The red lines and statistical indices refer to the mediating effect of decomposition on the relation between mental rotation and arithmetic skills. Analyses were controlled for age, sex, fluid and verbal reasoning. *** *p* < 0.001, ** *p* < 0.01, * *p* < 0.05

The indirect effect was significant for children’s usage of decomposition strategies, with *β* = 0.081, as indicated by a bootstrapped CI (0.017, 0.168). The indirect effects for the other arithmetic strategies were non-significant. This result indicates that the relation between children’s mental rotation and their arithmetic skills seems partially mediated by children’s usage of decomposition strategies (see Fig. 2). Children with higher mental rotation skills were more inclined to use decomposition strategies, which may in turn have helped them solving more arithmetic problems.

In addition to this multiple mediation model, we performed a number of post-hoc analyses to support and further probe the presence of a connection between mental rotation and arithmetic skills via decomposition. First, given the cross-sectional design of the present study, we also computed an additional multiple mediation model which was identical with respect to the general procedure and the included variables; however, in this reversed model, arithmetic skill was entered as predictor variable and mental rotation served as the criterion. Findings suggested that none of the indirect effects were significant, demonstrating no support for a mediating effect of decomposition skills in this reversed model. Second, in an additional mediation model, we added maternal education as an indicator of socio-economic status as another control variable but found no change in the results. The indirect effect of decomposition strategies was still significant, with *β* = 0.086, as indicated by a bootstrapped CI (0.016, 0.178). However, due to several missing values in this respective variable, the sample decreased to *n* = 174. Third, we investigated whether the indirect effect of decomposition strategies would emerge similarly in arithmetic problems involving a decade change and those without decade change. Considering that previous research showed that children tended to use more decomposition strategies in complex problems involving a decade change (e.g., Laski et al., 2014), it seems possible that the indirect effect of decomposition is stronger or only significant for those problems with decade change as opposed to easier problems without decade change. To answer this hypothesis, we categorized our arithmetic problems into problems involving a decade change and those without, resulting in 10 problems for each problem type. Then, we computed two scores of correct answers for each problem type, as well as the number of arithmetic strategies in each type (for descriptive statistics, see Table 5). As can be seen in Table 5, children were more accurate in solving problems without decade change as opposed to problems with decade change. In addition, they used more retrieval strategies in problems without decade change, and more decomposition strategies in problems with a decade change. Afterwards, we computed two additional multiple mediation models which were identical to the one above and used these scores as dependent variables, and the number of arithmetic strategies for the respective problem type as mediating variables. In the model with problems *with* decade change, the indirect effect was significant for children’s usage of decomposition strategies, with *β* = 0.091, as indicated by a bootstrapped CI (0.023, 0.189). In the model with problems *without* decade change, the indirect effect was lower but also significant for children’s usage of decomposition strategies, with *β* = 0.072, as indicated by a bootstrapped CI (0.002, 0.160). Therefore, it seems that the indirect effect linking children’s mental rotation and arithmetic skills holds for both problem types, irrespective of whether a decade change was involved in the arithmetic problem.

**Table 5:** Descriptive statistics for different types of mathematical problems (with and without decade change)

# correct	Type of mathematical problem				
	With decade change		Without decade change	*p *^*1,2*^	Cohen’s *d*
	6.49 (2.91)		7.38 (2.88)	< .001	.61
Arithmetic strategies					
Count-all	0.20 (0.90)		0.26 (0.90)	< .05	.16
Count-on	1.84 (2.67)		1.76 (2.58)	.422	− .06
Decomposition	**4.14 (3.46)**		**3.35 (3.09)**	** < .001**	− **.45**
Retrieval	**1.22 (1.93)**		**2.53 (2.59)**	** < .001**	**.84**

Data are mean (*SD*)

^1^To account for multiple testing, differences are interpreted as meaningful if the *p* values were significant after Bonferroni adjustment (*p* < 0.0125) and when the effect size was at least small

^2^Means were compared using *t*-tests

Bonferroni-corrected* p* values are presented in bold

### Differences between boys and girls? A closer look at interaction terms

Given that sex showed several correlations with the variables of interest, and in light of studies indicating differential relations between spatial and mathematical skills for girls and boys (e.g., Klein et al., 2010), we checked for interaction effects with sex in the multiple mediation model using model 8 and 14 of the PROCESS plug-in. Results indicated that none of the interactions were significant (all *t*s < 1.28, all *p*s > 0.20). Therefore, the present findings provided no support that sex moderated the relation between a) mental rotation and arithmetic skills, b) mental rotation and arithmetic strategies, and c) arithmetic strategies and arithmetic skills.

## Discussion

In the present study, we investigated whether children’s mental rotation and arithmetic skills were linked via their usage of arithmetic strategies. In line with previous research (Casey et al., 2017; Foley et al., 2017), it was found that children with higher mental rotation skills were more inclined to use higher-level mental strategies such as decomposition which in turn increased their accuracy of solving arithmetic problems. Notably, this result holds when controlling for maturational effects as reflected in age, but also when accounting for fluid and verbal reasoning. Overall, it seems that decomposition plays a unique role as an intervening process linking children’s mental rotation and arithmetic skills when considering that this respective strategy remained significant after simultaneously controlling for other strategies. This result can be interpreted such that spatial skills may help visualizing numerical magnitudes and increase flexibility in segmenting this magnitude into smaller parts. In turn, decomposing numbers is a powerful strategy to cope with complex arithmetic problems. Notably, this indirect effect did not emerge for the other mental strategy fact retrieval, highlighting that it is specifically the visualization and transformation process that is related to decomposition skills and not so much the memory process. If these associations would emerge just because memory skills are required in all our tasks, we would expect similar indirect relations via retrieval skills; however, mental rotation and retrieval were found to be unrelated to each other.

Moreover, this indirect effect emerged for arithmetic problems with decade change and those without decade change. In line with previous research (Laski et al., 2014), our results indicated that children used decomposition strategies more frequently in complex problems involving a decade change (when changing 15 + 8 to 15 + 5 + 3), even though it should be pointed out that they also decomposed numerical magnitudes in problems without decade change (when changing 12 + 7 to 12 + 3 + 4). This result does not necessarily undermine our main finding but rather shows that visualization and transformation processes are helpful for any kind of segmenting numerical magnitudes, no matter whether children would segment magnitudes along with the base-10 numerical system or in accordance with recently learnt number facts. Additionally, our results lend support to a number of previous studies showing that decomposition is closely related to mathematical achievement (Carr & Alexeev, 2011; Fennema et al., 1998; Geary et al., 2004). Overall, our findings accord to research showing close relations between girls’ spatial skills and their usage of decomposition (Casey et al., 2017; Laski et al., 2013) and extend this result to boys, highlighting that decomposition might be a universal intervening process in spatial-numerical associations.

In a set of follow-up multiple mediation models, we investigated whether boys and girls differed with respect to the strength of the relations among mental rotation, arithmetic strategies and calculation skills. However, our data did not yield any differences between girls and boys in the investigated relationships, as none of the interaction terms with sex were significant. Therefore, it seems that sex was not a moderating variable in the relations among mental rotation, arithmetic strategies, and arithmetic skills. Beyond these similarities in girls and boys, our results also showed some sex differences. Our findings suggested that girls were more inclined to use count-on strategies as opposed to higher-level mental strategies (decomposition and retrieval). Girls’ preference for counting strategies has been shown quite often in previous research (e.g., Carr & Davis, 2001; Carr et al., 2008; Fennema et al., 1998; Jordan et al., 2008), and seems to persist at least up to 5th grade (Imbo & Vandierendonck, 2007). Unfortunately, girls’ preference for counting strategies precludes leveraging opportunities to practice and improve more efficient higher-level mental strategies. Thus, this higher frequency of using counting strategies may constitute a risk factor for girls’ mathematical learning.

But why do girls and boys perform so differently in arithmetic strategy use? One explanation refers to girls’ difficulties in spatial reasoning (Casey & Fell, 2018; Laski et al., 2013). A robust male advantage in spatial skills and especially in mental rotation has been shown repeatedly and seems to increase with age (Miller & Halpern, 2013; for meta-analyses, see Linn & Petersen, 1985; Voyer et al., 1995). Even though this topic has been intensively investigated and discussed (e.g., Levine et al., 2016), as for today, it remains unknown why this difference emerges and persists (for a discussion, see Frick et al., 2014). With respect to our results, we see partial support for the hypothesis that girls’ lower frequency of higher-level strategies may refer to difficulties in spatial reasoning given that children’s mental rotation was indeed related to their usage of decomposition strategies. However, our findings did not support a sex difference in our chronometric mental rotation task, which is in line with findings of a recent meta-analysis, showing that a sex difference in mental rotation develops slowly across primary school (Lauer et al., 2019). Future studies may closely investigate this sex difference in strategy use and the role of spatial reasoning, with the goal to increase our in-depth understanding and provide equal opportunities for mathematical learning.

### Strengths and limitations

We consider it a strength of the present study that we have used widely acknowledged tasks which seemed to be suitable for children in the present age range. Furthermore, our sample size provided sufficient power in accordance to our power analysis, even though it would be preferrable to have an even larger sample size to investigate differential effects and interactions in the sample. Finally, we controlled for a number of influential variables that have been shown to influence children’s spatial and mathematical reasoning such as fluid reasoning (Atit et al., 2022; Peng et al., 2019), verbal ability (Kahl et al., 2021), as well as parental education (Carr et al., 2017; Möhring et al., 2021).

However, despite these strengths, it is also crucial to acknowledge the limitations of the present study when interpreting and drawing consequences from our results. First, the design was cross-sectional in nature. Even though we probed our findings by computing alternative, reversed models, it is crucial to replicate the present findings using a longitudinal design with several measurement time points. Second, our sample size spanned an age range from 6 to 8 years of age. Whereas decomposition might be an intervening process at this particular age, it seems possible that other (or multiple) intervening processes might be prominent at other ages. Future studies may examine a variety of intervening processes (including number line estimations, arithmetic strategies and others) and longitudinally track children to investigate multi-causal relations and see how these change with increasing age. Another limitation refers to the present chronometric mental rotation task. Even though this task allowed to assess whether children’s RTs and errors would be a linear function of the rotation angle, it would have been preferrable to assess additional spatial measures. Adding more measures from the intrinsic-dynamic category of spatial skills (cf. Newcombe & Shipley, 2015) would allow to create a latent variable and thus, a unified, error-free indicator of spatial skills. Additionally, including tasks from other categories in this typology would allow exploring whether decomposition relates specifically to the aspect of visualizing intrinsic properties or transforming them dynamically. Future studies may disentangle these aspects and investigate their specific relations to decomposition in particular and mathematics in general (for initial results, see Bates et al., 2020). A final limitation is that we did not videotape children’s mathematical performance, precluding a double-coding of arithmetic strategies, and determining inter-rater reliability.

### Educational implications

Findings of the present study can be transferred into a number of educational implications. First, the present study highlights once more that spatial thinking is an important cognitive ability when learning and succeeding in mathematics. Building upon evidence which emphasizes the malleability of spatial skills (for a meta-analysis, see Uttal et al., 2013) and far transfer from spatial training to math understanding (for a meta-analysis, see Hawes et al., 2022), future studies may focus on translating spatial thinking into classrooms and curricula (Gagnier & Fisher, 2020; Newcombe, 2010). Second, results of the present study add to our knowledge about intervening processes connecting space and mathematics. Findings can be helpful for intervention studies in order to disentangle the *specific* aspects in spatial trainings that are important for mathematical learning and may focus particularly on decomposition skills. Third, our results indicated differences in strategy use between males and females, with girls preferring counting strategies and using fewer higher-level mental strategies. The reasons thereof remain poorly understood with one explanation referring to spatial thinking (Casey & Fell, 2018; Laski et al., 2013). Whereas our findings represent an additional step in understanding this sex differences in strategy use, future studies may further scrutinize this difference and probe the role of spatial reasoning. For instance, intervention studies may particularly focus on how females’ (and males) strategy preferences change after training spatial skills.

## Data Availability

Data of the present study will be made publicly available via the Open Science Framework (OSF) and can be accessed at https://osf.io/dcsep/.

## Appendix

See Table

**Table 6:** Items of the mathematical task assessing arithmetic strategies

Item 1 (5 + 3)
Item 2 (7 + 8)
Item 3 (12 + 7)
Item 4 (15 + 8)
Item 5 (3 + 25)
Item 6 (9 + 26)
Item 7 (32 + 4)
Item 8 (37 + 6)
Item 9 (8 + 41)
Item 10 (2 + 49)
Item 11 (4 + 3 + 1 = _)
Item 12 (4 + _ = 5 + 3)
Item 13 (5 + 1 + 4 = _)
Item 14 (7 + _ = 4 + 5)
Item 15 (2 + 6 + 11 = _)
Item 16 (6 + _ = 12 + 9)
Item 17 (11 + 3 + 8 = _)
Item 18 (8 + _ = 26 + 5)
Item 19 (31 + 5 + 9 = _)
Item 20 (4 + _ = 33 + 5)

6.

## Footnotes

1 No information about handedness was available for 9 children.

2 Among other tasks, children also took part in a spatial scaling task, assessing children’s ability to relate spatial information between different-sized spatial layouts (cf. Möhring & Szubielska, 2023). Children’s accuracy in scaling spatial information was closely related to their mental rotation skills,* r*(177) = .23, *p* < .01, after accounting for age, sex, verbal and fluid reasoning. Therefore, it seems that these spatial abilities were closely connected. However, there was no relation between spatial scaling and mathematical performance, *r*(177) = -.13, *p* = .092, after accounting for control variables. Given that children took part in different perceptual conditions in the spatial scaling task due to a between-participants manipulation of perceptual condition, we decided to not include children’s spatial scaling in the present manuscript, despite previous work suggesting close relations between spatial scaling and mathematics (e.g., Frick, 2019; Gilligan et al., 2020; Möhring et al., 2018).

## References

[CR1] Atit, K., Power, J. R., Pigott, T., Lee, J., Geer, E. A., Uttal, D. H., Ganley, C. M., & Sorby, S. A. (2022). Examining the relations between spatial skills and mathematical performance: A meta-analysis. *Psychonomic Bulletin & Review,**29*(3), 699–720. 10.3758/s13423-021-02012-w 34799844 10.3758/s13423-021-02012-w

[CR2] Bailey, D. H. (2017). Causal inference and the spatial-math link in early childhood: Commentary. *Monographs of the Society for Research in Child Development,**82*(1), 127–136. 10.1111/mono.12288 28181250 10.1111/mono.12288

[CR3] Bates, K. E., Gilligan-Lee, K. A., & Farran, E. K. (2020). Reimagining mathematics: The role of mental imagery in explaining mathematical calculation skills in childhood. *Mind, Brain, and Education,**15*, 189–198. 10.1111/mbe.12281 10.1111/mbe.12281

[CR4] Canivez, G. L., & Watkins, M. W. (2016). Review of the Wechsler Intelligence Scale for Children-Fifth Edition: Critique, commentary, and independent analyses. In A. S. Kaufman, S. E. Raiford, & D. L. Coalson (Eds.), *Intelligent testing with the WISC-V* (pp. 683–702). Wiley.

[CR5] Carr, M., & Alexeev, N. (2011). Fluency, accuracy, and gender predict developmental trajectories of arithmetic strategies. *Journal of Educational Psychology,**103*(3), 617–631. 10.1037/a0023864 10.1037/a0023864

[CR6] Carr, M., & Davis, H. (2001). Gender differences in first-grade mathematics strategy use: A function of skills and preference. *Contemporary Education Psychology,**26*, 330–347. 10.1006/ceps.2000.1059 10.1006/ceps.2000.1059 11414724

[CR7] Carr, M., Hettinger Steiner, H. H., Kyser, B., & Biddlecomb, B. A. (2008). Comparison of predictors of early emerging gender differences in mathematics competency. *Learning and Individual Differences,**18*, 61–75. 10.1016/j.lindif.2007.04.005 10.1016/j.lindif.2007.04.005

[CR8] Carr, M., Alexeev, N., Wang, L., Barned, N., Horan, E., & Reed, A. (2017). The development of spatial skills in elementary school students. *Child Development,**89*, 446–460. 10.1111/cdev.12753 28186327 10.1111/cdev.12753

[CR82] Casey, B. & Ganley, C. (2021). An examination of gender differences in spatial skills and math attitudes in relation to mathematics success: A bio-psycho-social model. *Developmental Review, 60, *100963. 10.1016/j.dr.2021.100963 10.1016/j.dr.2021.100963

[CR9] Casey, M. B., Nuttall, R. L., Pezaris, E., & Benbow, C. P. (1995). The influence of spatial ability on gender differences in math college entrance test scores across diverse samples. *Developmental Psychology,**31*, 697–705. 10.1037/0012-1649.31.4.697 10.1037/0012-1649.31.4.697

[CR10] Casey, B. M., Lombardi, C. M., Pollock, A., Fineman, B., & Pezaris, E. (2017). Girls’ spatial skills and arithmetic strategies in first grade as predictors of fifth-grade analytical math reasoning. *Journal of Cognition and Development,**18*(5), 530–555. 10.1080/15248372.2017.1363044 10.1080/15248372.2017.1363044

[CR11] Casey, B. M., & Fell, H. (2018). Spatial Reasoning: A Critical Problem-Solving Tool in Children’s Mathematics Strategy Tool-Kit. In K. S. Mix & M. T. Battista (Eds.), *Visualizing Mathematics* (pp. 47–75). Springer International Publishing. 10.1007/978-3-319-98767-5_3

[CR12] Estes, D. (1998). Young children’s awareness of their mental activity: The case of mental rotation. *Child Development,**69*, 1345–1360. 10.1111/j.14678624.1998.tb06216.x 9839420 10.1111/j.14678624.1998.tb06216.x

[CR13] Faul, F., Erdfelder, E., Lang, A.-G., & Buchner, A. (2007). G*Power 3: A flexible statistical power analysis program for the social, behavioral, and biomedical sciences. *Behavior Research Methods,**39*(2), 175–191. 10.3758/BF03193146 17695343 10.3758/BF03193146

[CR14] Fennema, E., Carpenter, T. P., Jacobs, V. R., Franke, M. L., & Levi, L. W. (1998). A longitudinal study of gender differences in young children’s mathematical thinking. *Educational Researcher,**27*, 6–11.

[CR15] Foley, A. E., Vasilyeva, M., & Laski, E. V. (2017). Children’s use of decomposition strategies mediates the visuospatial memory and arithmetic accuracy relation. *British Journal of Developmental Psychology,**35*(2), 303–309. 10.1111/bjdp.12166 27966792 10.1111/bjdp.12166

[CR16] Frick, A. (2019). Spatial transformation abilities and their relation to later academic achievement. *Psychological Research Psychologische Forschung,**83*, 1465–1484. 10.1007/s00426-018-1008-5 29637258 10.1007/s00426-018-1008-5

[CR17] Frick, A., Möhring, W., & Newcombe, N. S. (2014). Development of mental transformation abilities. *Trends in Cognitive Sciences,**18*(10), 536–542. 10.1016/j.tics.2014.05.011 24973167 10.1016/j.tics.2014.05.011

[CR18] Fung, W. K., Chung, K. K. H., & Lam, C. B. (2020). Mathematics, executive functioning, and visual-spatial skills in Chinese kindergarten children: Examining the bidirectionality. *Journal of Experimental Child Psychology,**199*, 1–10. 10.1016/j.jecp.2020.104923 10.1016/j.jecp.2020.104923 32693935

[CR19] Gagnier, K. M., & Fisher, K. R. (2020). Unpacking the black box of translation: A framework for infusing spatial thinking into curricula. *Cognitive Research: Principles and Implications,**5*(1), 29. 10.1186/s41235-020-00222-9 32588283 10.1186/s41235-020-00222-9 PMC7316943

[CR20] Geary, D. C. (2011). Cognitive predictors of achievement growth in mathematics: A 5-year longitudinal study. *Developmental Psychology,**47*(6), 1539–1552. 10.1037/a0025510 21942667 10.1037/a0025510 PMC3210883

[CR21] Geary, D. C., Hoard, M. K., Byrd-Craven, J., & DeSoto, M. C. (2004). Strategy choices in simple and complex addition: Contributions of working memory and counting knowledge for children with mathematical disability. *Journal of Experimental Child Psychology,**88*(2), 121–151. 10.1016/j.jecp.2004.03.002 15157755 10.1016/j.jecp.2004.03.002

[CR22] Geer, E. A., Quinn, J. M., & Ganley, C. M. (2019). Relations between spatial skills and math performance in elementary school children: A longitudinal investigation. *Developmental Psychology,**55*(3), 637–652. 10.1037/dev0000649 30550325 10.1037/dev0000649

[CR23] Gilligan, K. A., Thomas, M. S. C., & Farran, E. K. (2020). First demonstration of effective spatial training for near transfer to spatial performance and far transfer to a range of mathematics skills at 8 years. *Developmental Science*. 10.1111/desc.12909 31599470 10.1111/desc.12909 PMC7379338

[CR24] Groth-Marnat, G. (2009). *Handbook of psychological assessment* (5th ed.). John Wiley & Sons, Inc.

[CR25] Gunderson, E. A., & Hildebrand, L. (2021). Relations among spatial skills, number line estimation, and exact and approximate calculation in young children. *Journal of Experimental Child Psychology*. 10.1016/j.jecp.2021.105251 34333360 10.1016/j.jecp.2021.105251

[CR26] Gunderson, E. A., Ramirez, G., Beilock, S. L., & Levine, S. C. (2012). The relation between spatial skill and early number knowledge: The role of the linear number line. *Developmental Psychology,**48*(5), 1229–1241. 10.1037/a0027433 22390659 10.1037/a0027433 PMC10811729

[CR27] Hawes, Z., Gilligan-Lee, K. A., & Mix, K. S. (2022). Effects of spatial training on mathematics performance: A meta-analysis. *Developmental Psychology,**58*(1), 112–137. 10.1037/dev0001281 35073120 10.1037/dev0001281

[CR28] Hayes, A. F. (2018). *Introduction to mediation, moderation, and conditional process analysis: A regression-based approach*. Guilford Press.

[CR29] Hespos, S. J., & Rochat, P. (1997). Dynamic mental representation in infancy. *Cognition,**64*(2), 153–188. 10.1016/s0010-0277(97)00029-2 9385869 10.1016/s0010-0277(97)00029-2

[CR30] Imbo, I., & Vandierendonck, A. (2007). The development of strategy use in elementary school children: Working memory and individual differences. *Journal of Experimental Child Psychology,**96*(4), 284–309. 10.1016/j.jecp.2006.09.001 17046017 10.1016/j.jecp.2006.09.001

[CR31] Ishihara, M., Jacquin-Courtois, S., Flory, V., Salemme, R., Imanaka, K., & Rossetti, Y. (2006). Interaction between space and number representations during motor preparation in manual aiming. *Neuropsychologia,**44*, 1009–1016. 10.1016/j.neuropsychologia.2005.11.008 16406028 10.1016/j.neuropsychologia.2005.11.008

[CR32] Jordan, N. C., Kaplan, D., Ramineni, C., & Locuniak, M. N. (2008). Development of number combination skill in the early school years: When do fingers help? *Developmental Science,**11*(5), 662–668. 10.1111/j.1467-7687.2008.00715.x 18801121 10.1111/j.1467-7687.2008.00715.x

[CR33] Judd, N., & Klingberg, T. (2021). Training spatial cognition enhances mathematical learning in a randomized study of 17,000 children. *Nature Human Behaviour,**5*(11), 1548–1554. 10.1038/s41562-021-01118-4 34017098 10.1038/s41562-021-01118-4

[CR34] Kahl, T., Grob, A., Segerer, R., & Möhring, W. (2021). Executive functions and visual-spatial skills predict mathematical achievement: Asymmetrical associations across age. *Psychological Research Psychologische Forschung,**85*, 36–46. 10.1007/s00426-019-01249-4 31560097 10.1007/s00426-019-01249-4

[CR35] Kahl, T., Segerer, R., Grob, A., & Möhring, W. (2022). Bidirectional associations among executive functions, visual-spatial skills, and mathematical achievement in primary school students: Insights from a longitudinal study. *Cognitive Development*. 10.1016/j.cogdev.2021.101149 10.1016/j.cogdev.2021.101149

[CR36] Kell, H. J., Lubinski, D., Benbow, C. P., & Steiger, J. H. (2013). Creativity and technical innovation: Spatial ability’s unique role. *Psychological Science,**24*(9), 1831–1836. 10.1177/0956797613478615 23846718 10.1177/0956797613478615

[CR37] Klein, P. S., Adi-Japha, E., & Hakak-Benizri, S. (2010). Mathematical thinking of kindergarten boys and girls: Similar achievement, different contributing processes. *Educational Studies in Mathematics,**73*, 233–246. 10.1007/s10649-009-9216-y 10.1007/s10649-009-9216-y

[CR38] Kosslyn, S. M. (1975). Information representation in visual images. *Cognitive Psychology,**7*(3), 341–370. 10.1016/0010-0285(75)90015-8 10.1016/0010-0285(75)90015-8

[CR39] Kosslyn, S. M., Margolis, J. A., Barrett, A. M., Goldknopf, E. J., & Daly, P. F. (1990). Age differences in imagery abilities. *Child Development,**61*(4), 995–1010. 2209202 10.2307/1130871

[CR40] Kyttälä, M., & Lehto, J. E. (2008). Some factors underlying mathematical performance: The role of visuospatial working memory and non-verbal intelligence. *European Journal of Psychology of Education,**23*(1), 77–94. 10.1007/BF03173141 10.1007/BF03173141

[CR42] Laski, E. V., Casey, B. M., Yu, Q., Dulaney, A., Heyman, M., & Dearing, E. (2013). Spatial skills as a predictor of first grade girls’ use of higher level arithmetic strategies. *Learning and Individual Differences,**23*, 123–130. 10.1016/j.lindif.2012.08.001 10.1016/j.lindif.2012.08.001

[CR43] Laski, E. V., Ermakova, A., & Vasilyeva, M. (2014). Early use of decomposition for addition and its relation to base-10 knowledge. *Journal of Applied Developmental Psychology,**35*(5), 444–454. 10.1016/j.appdev.2014.07.002 10.1016/j.appdev.2014.07.002

[CR44] Lauer, J. E., & Lourenco, S. F. (2016). Spatial processing in infancy predicts both spatial and mathematical aptitude in childhood. *Psychological Science,**27*(10), 1291–1298. 10.1177/0956797616655977 27528464 10.1177/0956797616655977

[CR45] Lauer, J. E., Yhang, E., & Lourenco, S. F. (2019). The development of gender differences in spatial reasoning: A meta-analytic review. *Psychological Bulletin,**145*(6), 537–565. 10.1037/bul0000191 30973235 10.1037/bul0000191

[CR46] LeFevre, J.-A., Jimenez Lira, C., Sowinski, C., Cankaya, O., Kamawar, D., & Skwarchuk, S.-L. (2013). Charting the role of the number line in mathematical development. *Frontiers in Psychology,**4*, 641. 10.3389/fpsyg.2013.00641 24065943 10.3389/fpsyg.2013.00641 PMC3776572

[CR47] Levine, S. C., Foley, A., Lourenco, S., Ehrlich, S., & Ratliff, K. (2016). Sex differences in spatial cognition: Advancing the conversation. *Wiley Interdisciplinary Reviews. Cognitive Science,**7*(2), 127–155. 10.1002/wcs.1380 26825049 10.1002/wcs.1380

[CR48] Linn, M. C., & Petersen, A. C. (1985). Emergence and characterization of sex differences in spatial ability: A meta-analysis. *Child Development,**56*(6), 1479–1498. 10.1111/j.1467-8624.1985.tb00213 4075870 10.1111/j.1467-8624.1985.tb00213

[CR49] Lohman, D. F. (1996). Spatial ability and g. In I. Dennis & P. Tapsfield (Eds.), *Human abilities: Their nature and measurement* (pp. 97–116). Lawrence Erlbaum Associates Inc.

[CR50] Lowrie, T., Logan, T., & Ramful, A. (2017). Visuospatial training improves elementary students’ mathematics performance. *British Journal of Educational Psychology,**87*(2), 170–186. 10.1111/bjep.12142 28097646 10.1111/bjep.12142

[CR51] Lowrie, T., Resnick, I., Harris, D., & Logan, T. (2020). In search of the mechanisms that enable transfer from spatial reasoning to mathematics understanding. *Mathematics Education Research Journal,**32*(2), 175–188. 10.1007/s13394-020-00336-9 10.1007/s13394-020-00336-9

[CR52] Marmor, G. S. (1975). Development of kinetic images: When does the child first represent movement in mental images? *Cognitive Psychology,**7*, 548–559. 10.1016/0010-0285(75)90022-5 10.1016/0010-0285(75)90022-5

[CR53] Miller, D. I., & Halpern, D. (2013). The new science of cognitive sex differences. *Trends in Cognitive Sciences,**18*, 37–45. 10.1016/j.tics.2013.10.011 24246136 10.1016/j.tics.2013.10.011

[CR54] Mix, K. S. (2019). Why Are Spatial Skill and Mathematics Related? *Child Development Perspectives,**13*(2), 121–126. 10.1111/cdep.12323 10.1111/cdep.12323

[CR55] Mix, K. S., Levine, S. C., Cheng, Y.-L., Young, C., Hambrick, D. Z., Ping, R., & Konstantopoulos, S. (2016). Separate but correlated: The latent structure of space and mathematics across development. *Journal of Experimental Psychology: General,**145*(9), 1206–1227. 10.1037/xge0000182 27560854 10.1037/xge0000182

[CR56] Mix, K. S., & Cheng, Y.-L. (2012). The relation between space and math. In J. B. Benson (Ed.), *Advances in Child Development and Behavior* (pp. 197–243). 10.1016/B978-0-12-394388-0.00006-X 10.1016/b978-0-12-394388-0.00006-x 22675907

[CR57] Moè, A. (2018). Mental rotation and mathematics: Gender-stereotyped beliefs and relationships in primary school children. *Learning and Individual Differences,**61*, 172–180. 10.1016/j.lindif.2017.12.002 10.1016/j.lindif.2017.12.002

[CR58] Möhring, W., & Frick, A. (2013). Touching up mental rotation: Effects of manual experience on 6-month-old infants’ mental object rotation. *Child Development,**84*(5), 1554–1565. 10.1111/cdev.12065 23432700 10.1111/cdev.12065

[CR59] Möhring, W., & Szubielska, M. (2023). Scaling up = scaling down? Children’s spatial scaling in different perceptual modalities and scaling directions. *Cognitive Research: Principles and Implications,**8*(1), 62. 10.1186/s41235-023-00517-7 37794290 10.1186/s41235-023-00517-7 PMC10550888

[CR60] Möhring, W., Frick, A., & Newcombe, N. S. (2018). Spatial scaling, proportional thinking, and numerical understanding in 5- to 7-year-old children. *Cognitive Development,**45*, 57–67. 10.1016/j.cogdev.2017.12.001 10.1016/j.cogdev.2017.12.001

[CR61] Möhring, W., Ishihara, M., Curiger, J., & Frick, A. (2019). Spatial-numerical associations in 1^st^-graders: Evidence from a manual-pointing task. *Psychological Research Psychologische Forschung,**83*, 885–893. 10.1007/s00426-017-0904-4 28799042 10.1007/s00426-017-0904-4

[CR62] Möhring, W., Ribner, A. D., Segerer, R., Libertus, M. E., Kahl, T., Troesch, L. M., & Grob, A. (2021). Developmental trajectories of children’s spatial skills: Influencing variables and associations with later mathematical thinking. *Learning and Instruction*. 10.1016/j.learninstruc.2021.101515 10.1016/j.learninstruc.2021.101515

[CR63] Moore, D. S., & Johnson, S. P. (2008). Mental rotation in human infants: A sex difference. *Psychological Science,**19*(11), 1063–1066. 10.1111/j.1467-9280.2008.02200.x 19076473 10.1111/j.1467-9280.2008.02200.x PMC2651884

[CR64] Newcombe, N. S. (2010). Picture this: Increasing math and science learning by improving spatial thinking. *American Educator,**34*, 29–43.

[CR65] Newcombe, N., & Shipley, T. (2015). Thinking About Spatial Thinking: New Typology, New Assessments. In J. Gero (Ed.) *Studying Visual and Spatial Reasoning for Design Creativity.* Springer. 10.1007/978-94-017-9297-4_10

[CR66] Newcombe, N. S., Möhring, W., & Frick, A. (2018). How big is many? Development of spatial and numerical magnitude understanding. In A. Henik & W. Fias (Eds.), *Heterogeneity of function in numerical cognition* (pp. 157–176). 10.1016/B978-0-12-811529-9.00009-1

[CR67] Peng, P., Wang, T., Wang, C., & Lin, X. (2019). A Meta-analysis on the relation between fluid intelligence and reading/mathematics: Effects of tasks, age, and social economics status. *Psychological Bulletin,**145*, 189–236. 10.1037/bul0000182 30652909 10.1037/bul0000182

[CR68] Perrucci, V., Agnoli, F., & Albiero, P. (2008). Children’s performance in mental rotation tasks: Orientation-free features flatten the slope. *Developmental Science,**11*, 732–742. 10.1111/j.1467-7687.2008.00723.x 18801129 10.1111/j.1467-7687.2008.00723.x

[CR69] Petermann, F. (2017). *Wechsler Intelligence Scales for Children: Fifth Edition* (WISC-V; German Version). Pearson Assessment.

[CR70] Quinn, P. C., & Liben, L. S. (2008). A sex difference in mental rotation in young infants. *Psychological Science,**19*(11), 1067–1070. 10.1111/j.1467-9280.2008.02201.x 19076474 10.1111/j.1467-9280.2008.02201.x

[CR71] Ramirez, G., Chang, H., Maloney, E. A., Levine, S. C., & Beilock, S. L. (2016). On the relationship between math anxiety and math achievement in early elementary school: The role of problem solving strategies. *Journal of Experimental Child Psychology,**141*, 83–100. 10.1016/j.jecp.2015.07.014 26342473 10.1016/j.jecp.2015.07.014

[CR72] Schwarzer, G., Freitag, C., Buckel, R., & Lofruthe, A. (2013). Crawling is associated with mental rotation ability by 9-month-old infants. *Infancy,**18*(3), 432–441. 10.1111/j.1532-7078.2012.00132.x 10.1111/j.1532-7078.2012.00132.x

[CR73] Shepard, R. N., & Metzler, J. (1971). Mental rotation of three-dimensional objects. *Science,**171*, 701–703. 10.1126/science.171.3972.701 5540314 10.1126/science.171.3972.701

[CR74] Shrager, J., & Siegler, R. S. (1998). SCADS: A model of children’s strategy choices and strategy discoveries. *Psychological Science,**9*, 405–410. 10.1111/1467-9280.00076 10.1111/1467-9280.00076

[CR75] Uttal, D. H., Meadow, N. G., Tipton, E., Hand, L. L., Alden, A. R., Warren, C., & Newcombe, N. S. (2013). The malleability of spatial skills: A meta-analysis of training studies. *Psychological Bulletin,**139*(2), 352–402. 10.1037/a0028446 22663761 10.1037/a0028446

[CR76] Verdine, B. N., Golinkoff, R. M., Hirsh-Pasek, K., & Newcombe, N. S. (2017). Spatial skills, their development, and their links to mathematics. *Monographs of the Society for Research in Child Development,**82*(1), 7–30. 10.1111/mono.12280 28181248 10.1111/mono.12280

[CR77] Voyer, D., Voyer, S., & Bryden, M. P. (1995). Magnitude of sex differences in spatial abilities: A meta-analysis and consideration of critical variables. *Psychological Bulletin,**117*(2), 250–270. 10.1037/0033-2909.117.2.250 7724690 10.1037/0033-2909.117.2.250

[CR78] Wai, J., Lubinski, D., & Benbow, C. P. (2009). Spatial ability for STEM domains: Aligning over 50 years of cumulative psychological knowledge solidifies its importance. *Journal of Educational Psychology,**101*(4), 817–835. 10.1037/a0016127 10.1037/a0016127

[CR79] Wechsler, D. (2014). *Wechsler Intelligence Scales for Children: Fifth Edition (WISC-V).* Pearson.

[CR80] Xie, F., Zhang, L., Chen, X., & Xin, Z. (2020). Is spatial ability related to mathematical ability: A meta-analysis. *Educational Psychology Review,**32*(1), 113–155. 10.1007/s10648-019-09496-y 10.1007/s10648-019-09496-y

[CR81] Young, C. J., Levine, S. C., & Mix, K. S. (2018). The connection between spatial and mathematical ability across development. *Frontiers in Psychology,**9*, 755. 10.3389/fpsyg.2018.00755 29915547 10.3389/fpsyg.2018.00755 PMC5994429

